# Neuroimaging characteristics associated with early neurological deterioration in single subcortical infarction patients

**DOI:** 10.3389/fneur.2026.1849475

**Published:** 2026-08-05

**Authors:** Yating Wu, Le Chen, Zezheng Sun, Jingtao Pi, Jian Wu

**Affiliations:** 1 Department of Neurology, Beijing Tsinghua Changgung Hospital, School of Clinical Medicine, Tsinghua Medicine, Tsinghua University, Beijing, China; 2 IDG/McGovern Institute for Brain Research at Tsinghua University, Beijing, China

**Keywords:** ADC, CSVD, early neurological deterioration, plaque, single subcortical infarction

## Abstract

**Background and purpose:**

Single subcortical infarctions (SSIs) are frequently complicated by early neurological deterioration (END), which adversely impacts long-term functional outcomes. However, the neuroimaging predictors of END in SSI patients remain insufficiently characterized. This study aimed to comprehensively evaluate the predictive value of multiple neuroimaging biomarkers for END in SSI patients.

**Methods:**

We retrospectively enrolled consecutive patients diagnosed with acute SSIs in the middle cerebral artery (MCA) perforating territory. Neuroimaging assessments included apparent diffusion coefficient (ADC) and relative ADC (rADC) values, axial infarct location, MCA plaque status evaluated by whole-brain vessel wall imaging, and cerebral small vessel disease (CSVD) burden. END was defined as an increase of ≥2 points in the total NIHSS score or ≥1 point in the motor subscore within 72 h of admission.

**Results:**

A total of 129 SSI patients were enrolled, of whom 32 (24.8%) developed END. Lower ADC and rADC values, larger maximum infarct diameter, more proximal axial infarct location, and the presence of MCA plaques were significantly associated with END on univariate analysis. rADC demonstrated superior predictive performance over ADC, with an optimal cut-off of 0.61 (sensitivity 84%, specificity 50%). On multivariable logistic regression, rADC < 0.61 (OR = 4.23, 95% CI: 1.58–11.61, *P* = 0.004) and MCA plaque presence (OR = 2.80, 95% CI: 1.14–6.89, *P* = 0.025) emerged as independent predictors of END. Neither CSVD burden nor any individual CSVD imaging marker was significantly associated with END.

**Conclusions:**

rADC and MCA plaque are independent neuroimaging predictors of END in SSI patients, while CSVD burden showed no significant association. These findings support early magnetic resonance imaging-based risk stratification and individualized therapeutic decision-making in clinical practice.

## Introduction

Infarctions occurring in the territory of perforating arteries are defined as single subcortical infarctions (SSIs). Compared with other subtypes of ischemic stroke, patients with SSIs more frequently experience symptom fluctuations (1, 2). Approximately 13%−47.5% of SSI patients may develop early neurological deterioration (END), which adversely affects long-term functional prognosis (3, 4). Therefore, identifying patients at high-risk for END is of critical importance, as it enables timely risk assessment and targeted therapeutic intervention in clinical practice.

It has been demonstrated that SSI result from two distinct etiological mechanisms: lipohyalinosis or fibrinoid degeneration of the distal perforating arteries, characteristic of cerebral small vessel disease (CSVD), and atherosclerotic occlusion at the proximal perforating artery orifice attributable to parent artery plaque or microatheroma formation, the latter known as branch atheromatous disease (BAD) (5–7). This pathophysiological heterogeneity is intimately reflected in the neuroimaging features of SSI, which may thereby serve as potential indicators of END risk.

CSVD burden has been identified as a predictor of stroke outcomes (8, 9); however, its specific relationship with END remains controversial (10). Although the magnitude of apparent diffusion coefficient (ADC) reduction has been linked to clinical and tissue outcomes in territorial infarctions (11, 12), its predictive value for END in SSI patients remains underexplored. Moreover, prior studies have confirmed that distinct spatial lesion patterns are closely associated with the development of END (13, 14), but most have relied on simple binary classification of SSIs into proximal and distal subtypes (13, 15), lacking quantitative indices of infarct location on imaging. More recently, whole-brain vessel wall imaging (WB-VWI) has enabled direct visualization of parent artery plaques, offering a more refined approach to END risk assessment in SSI patients. Therefore, the present study aimed to comprehensively evaluate the predictive value of magnetic resonance imaging (MRI) characteristics, including CSVD burden, ADC values, infarct topography, parent artery plaque status, and other MRI-derived imaging markers, for END in SSI patients, with the goal of facilitating early risk stratification in clinical practice.

## Materials and methods

### Study population

We performed a retrospective analysis of consecutive patients diagnosed with SSIs in the middle cerebral artery (MCA) territory at Beijing Tsinghua Changgung Hospital between January 2017 and December 2024. All enrolled participants satisfied the following criteria: (1) age ≥18 years, (2) admitted within 72 h of symptom onset, (3) comprehensive stroke evaluations were conducted, including MRI, magnetic resonance angiography (MRA), whole-brain vessel wall imaging (WB-VWI), echocardiography, electrocardiography (ECG), and laboratory examinations. Patients were excluded if they had: (1) potential cardioembolic sources, or (2) significant ipsilateral intracranial or extracranial arterial stenosis (>50%) (13, 16, 17). The ethics committee of Beijing Tsinghua Changgung Hospital approved the protocol for this study.

### Clinical information

Demographic characteristics, clinical parameters, and vascular risk factors were collected, which encompassed age, sex, hypertension, diabetes mellitus, hyperlipidemia, smoking history, prior stroke history, and the National Institutes of Health Stroke Scale (NIHSS) scores at admission. Laboratory data obtained during hospitalization included blood glucose and lipid profiles. END was defined as an increase of ≥2 points in the total NIHSS score or ≥1 point in the motor subscore within 72 h of admission, relative to the baseline assessment (18, 19).

### Imaging protocol

Brain MRI was conducted for all enrolled patients within 7 days after admission. Conventional MRI sequences, including T1WI, T2WI, FLAIR, DWI, and ADC maps, were obtained as early as possible after symptom onset to assess infarct characteristics. The WB-VWI protocol, which was used for plaque assessment, included a 3D time-of-flight (TOF) MRA and 3D T1-volume isotropic turbo spin-echo acquisition (VISTA) or CUBE T1-weighted imaging sequences. MRI was performed on 3.0-tesla systems (Discovery 750, GE HealthCare, Chicago, IL, USA; Ingenia CX, Philips Healthcare, Best, the Netherlands). Common 3D TOF MRA was performed: repetition time (TR)/ echo time (TE) 22/2.4 ms; flip angle 20°; slice thickness 1.1 mm. The scan parameters for CUBE T1-weighted imaging sequences were as follows: TR/TE 800/16 ms, flip angle 90°, slice thickness 0.6 mm, field of view (FOV) 23 × 18.4 cm^2^, and spatial resolution 0.7 × 0.6 × 0.6 mm^3^. The parameters for 3D T1-VISTA sequence were as follows: TR/TE 600/32 ms, flip angle 90°, slice thickness 1.1 mm, FOV 4.5 × 20 × 20 cm^3^; spatial resolution 0.6 × 0.6 × 0.6 mm^3^.

### Radiological assessment

**Figure 1:**
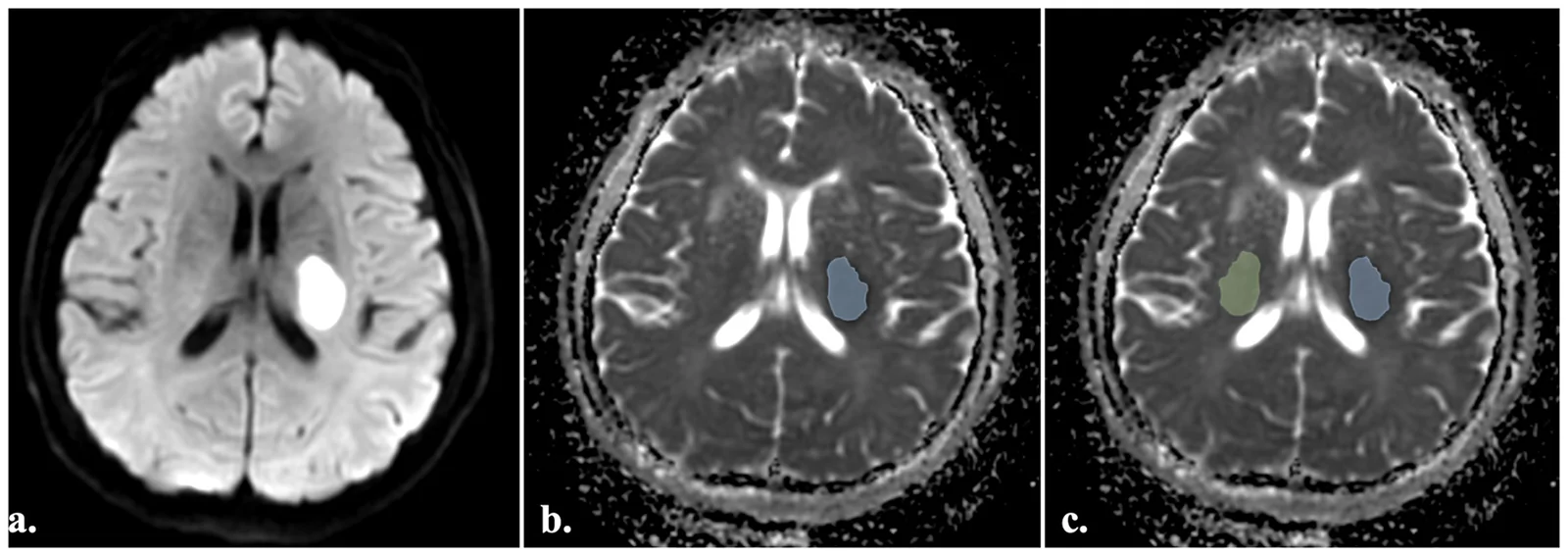
Representative measurement of ADC and rADC values. **(a)** DWI showing the largest infarct layer. **(b)** A ROI was manually placed within the ischemic lesion on the corresponding ADC map to measure the ADC value. **(c)** A mirror ROI was placed in the corresponding contralateral, non-ischemic hemisphere. The rADC was calculated as the ratio of the mean ADC value of the ischemic ROI to that of the contralateral mirror ROI.

All MR images were transferred to a digital picture archiving and communication system (PACS) workstation for analysis. Based on DWI, the maximum infarct area and maximum infarct diameter were measured on axial slices. Infarct volume was calculated by multiplying the slice thickness (5 mm) by the sum of infarct areas across all DWI layers. The largest infarct layer was selected as the region of interest (ROI), and ADC values were measured manually within this region. The relative ADC (rADC) was subsequently calculated as the ratio of the mean ADC value of the ischemic ROI to that of the mirror ROI in the contralateral, non-ischemic hemisphere (Figure 1). All of the above quantitative imaging measurements were performed by an experienced neurologist who was blinded to clinical information and END status. CSVD burden was independently assessed by two neurologists using a validated four-item scoring system (total score: 0–4), with 1 point assigned for each of the following: lacunar infarction (≥1 lacune), any microbleeds (CMB), moderate-to-severe enlarged perivascular spaces (EPVS) in the basal ganglia (grade 2–4), deep white matter hyperintensities (dWMH; Fazekas grade 2–3), or periventricular white matter hyperintensities (pWMH; Fazekas grade 3) (20, 21).

**Figure 2:**
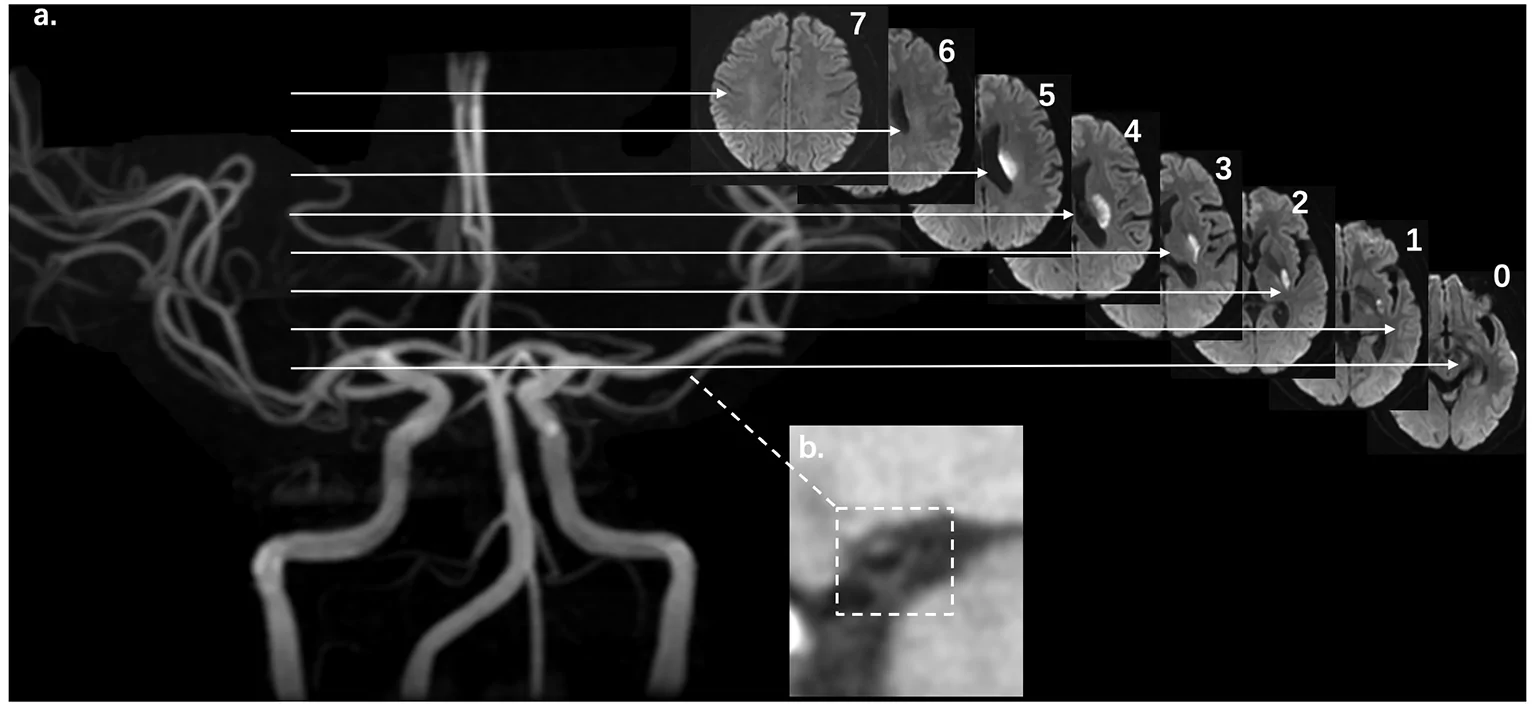
Schematic diagram of evaluating the lowest infarct layer index (LILI) and MCA plaque. **(a)** The left side is the coronal 3D TOF MRA image, and the right side is the DWI images. Layer 0 is the index for the axial layer of DWI at the circle of Willis level. Next, the first layer's index indicating deep subcortical infarction is LILI (arrowhead). This figure shows a patient with LILI = 1. **(b)**WB-VWI image showed the presence of MCA plaque.

To quantify the longitudinal infarct location, the lowest infarct layer index (LILI) was recorded for each enrolled SSI patient as previous studies (22, 23). The level of the circle of Willis on time-of-flight (TOF) imaging and the MCA flow void on T2-weighted imaging (T2WI) were used as reference points, with the corresponding DWI layer designated as layer 0. Subsequent layers were indexed sequentially from the proximal deep subcortical region toward the distal branch territory, with an inter-slice interval of 5 mm. MCA plaques were identified as focal or asymmetric wall thickening of the M1 segment situated near the origin of the lenticulostriate artery (LSA) by WB-VWI, and plaque location as well as its spatial relationship with the infarct were evaluated on both short -axial and long-axial images (Figure 2).

### Statistical analysis

Continuous variables with normal distribution were expressed as mean ± standard deviation, and those with non-normal distributed as median (interquartile range). Categorical variables were presented as counts and percentages, and compared using the χ^2^ test or Fisher's exact test. Between-group differences in continuous variables were assessed using the independent samples *t*-test for normally distributed data and the Mann–Whitney *U* test for non-normally distributed data. Multivariable logistic regression analysis was performed to identify independent predictors of END, incorporating age, sex, and all variables with *P* < 0.05 in univariable analyses. Receiver operating characteristic (ROC) curve analysis was performed to evaluate the predictive performance of ADC and rADC for END, and the optimal cutoff value was determined using the Youden index. All data analyses were conducted by SPSS 25.0 (IBM, New York, USA). A two-tailed *P*-value < 0.05 was considered statistically significant.

## Results

### Patients' characteristics

Between January 2017 and December 2024, 129 patients with acute SSIs in the perforating territory of the MCA were enrolled, including 83 (64.3%) males and 46 (35.7%) females. The mean age at onset of SSI was 61.10 ± 11.42 years. Hypertension was the most prevalent vascular risk factor, identified in 88 patients (68.2%), followed by smoking history in 58 (45.0%), diabetes mellitus in 47 (36.4%), hyperlipidemia in 36 (27.9%), and prior stroke in 29 (22.5%). END occurred in 32 patients (24.8%) within 72 h of admission. Of these, 12 patients (37.5%) developed END within 24 h. Regarding initial acute-phase treatments, 19 patients (14.7%) received intravenous thrombolysis, 52 (40.3%) received dual antiplatelet therapy, and 77 (59.7%) received mono antiplatelet therapy. Baseline demographic characteristics, vascular risk factors, laboratory data and initial acute-phase treatments were comparable between the END and non-END groups (Table 1).

**Table 1:** Baseline characteristics of groups with and without END.

Characteristics	No-END (97)	END (32)	*P-*value
Age (mean ± SD)	60.84 ± 11.17	61.91 ± 12.29	0.647
Sex, male, *n* (%)	62 (63.9%)	19 (59.4%)	0.645
Risk factors			
Hypertension, *n* (%)	63 (65.0%)	25 (78.1%)	0.165
Diabetes, *n* (%)	37 (38.1%)	10 (31.3%)	0.482
Hyperlipidemia, *n* (%)	29 (29.9%)	7 (21.9%)	0.364
Smoking history, *n* (%)	58 (59.8%)	14 (43.8%)	0.371
Previous stroke, *n* (%)	25 (25.8%)	4 (12.5%)	0.147
Initial NIHSS score, median (IQR)	2.5 (2–5)	3 (2–5)	0.808
Laboratory data			
Blood glucose, mmol/L, median (IQR)	7.72 (6.04–10.9)	7.70 (6.47–10.64)	0.900
Total cholesterol, mg/dL (mean ± SD)	4.55 ± 0.98	4.62 ± 1.04	0.763
LDL cholesterol, mg/dL (mean ± SD)	2.90 ± 0.83	2.95 ± 0.95	0.788
HDL cholesterol, mg/dL, median (IQR)	1.04 ± 0.26	1.02 ± 0.33	0.751
Triglyceride, mg/dL, median (IQR)	1.47 (1.06–2.21)	1.42 (1.13–1.69)	0.399
Initial acute-phase treatment			
Intravenous thrombolysis, *n* (%)	11 (11.3%)	8 (25.0%)	0.082
Antiplatelet therapy, *n* (%)			0.383
Dual antiplatelet therapy	37 (38.1%)	15 (46.9%)	
Mono antiplatelet therapy	60 (61.9%)	17 (53.1%)	

LDL, low density lipoprotein; HDL, high density lipoprotein; SD, standard deviation; IQR, interquartile range; NIHSS, National Institutes of Health Stroke Scale.

### Neuroimaging characteristics in SSI patients

The median LILI was 2 (IQR: 2–3). MCA plaque was identified in 47 (36.3%) patients. Table 2 summarizes the radiological characteristics of the study population. In the univariate analysis, both ADC and rADC values were significantly lower in the END group compared with the non-END group. END was also significantly associated with maximum infarct diameter, LILI value, and the presence of MCA plaques. In contrast, neither the total CSVD score nor any individual CSVD imaging marker, including lacunes, WMH, CMBs, and EPVS showed a significant association with END in SSI patients.

**Table 2:** Neuroimaging characteristics of groups with and without END.

Neuroimaging characteristics	No-END (97)	END (32)	*P-*value
Infarct volume, cm^3^, median (IQR)	1.04 (0.49–2.45)	1.39 (0.59–2.95)	0.216
Maximum infarct area on axial, cm^2^, median (IQR)	0.96 (0.52–1.76)	1.23 (0.77–2.31)	0.070
Maximum infarct diameter on axial, cm, median (IQR)	1.42 (1.08–2.04)	1.84 (1.39–2.20)	0.012^*^
The number of infarct slices, (mean ± SD)	4.40 ± 1.50	4.41 ± 1.50	0.989
LILI, median (IQR)	2 (2–3)	2 (1–2)	0.038^*^
MCA plaques, *n* (%)	30 (30.9%)	19 (59.4%)	0.004^*^
ADC of ischemic voxels, (mean ± SD)	487.27 ± 71.58	453.22 ± 70.02	0.021^*^
rADC of ischemic voxels, median (IQR)	0.61 (0.56–0.70)	0.55 (0.51–0.60)	< 0.001^*^
Total CSVD score, median (IQR)	1 (0–2)	1 (0–2)	0.595
Lacune, *n* (%)	49 (50.5%)	14 (43.8%)	0.507
pWMH, median (IQR)	1 (1–2)	1 (1–3)	0.576
dWMH, median (IQR)	1 (1–2)	1 (0–2)	0.754
CMB, *n* (%)	28 (28.9%)	8 (25.0%)	0.672
EPVS, *n* (%)	17 (17.5%)	3 (9.4%)	0.269

SD, standard deviation; IQR, interquartile range; LILI, the lowest infarct layer index; ADC, apparent diffusion coefficient; MCA, middle cerebral artery; CSVD, cerebral small vessel disease; CMB, microbleeds; EPVS, enlarged perivascular spaces; dWMH, deep white matter hyperintensities; pWMH, periventricular white matter hyperintensities.

^*^Indicates statistical significance.

**Figure 3:**
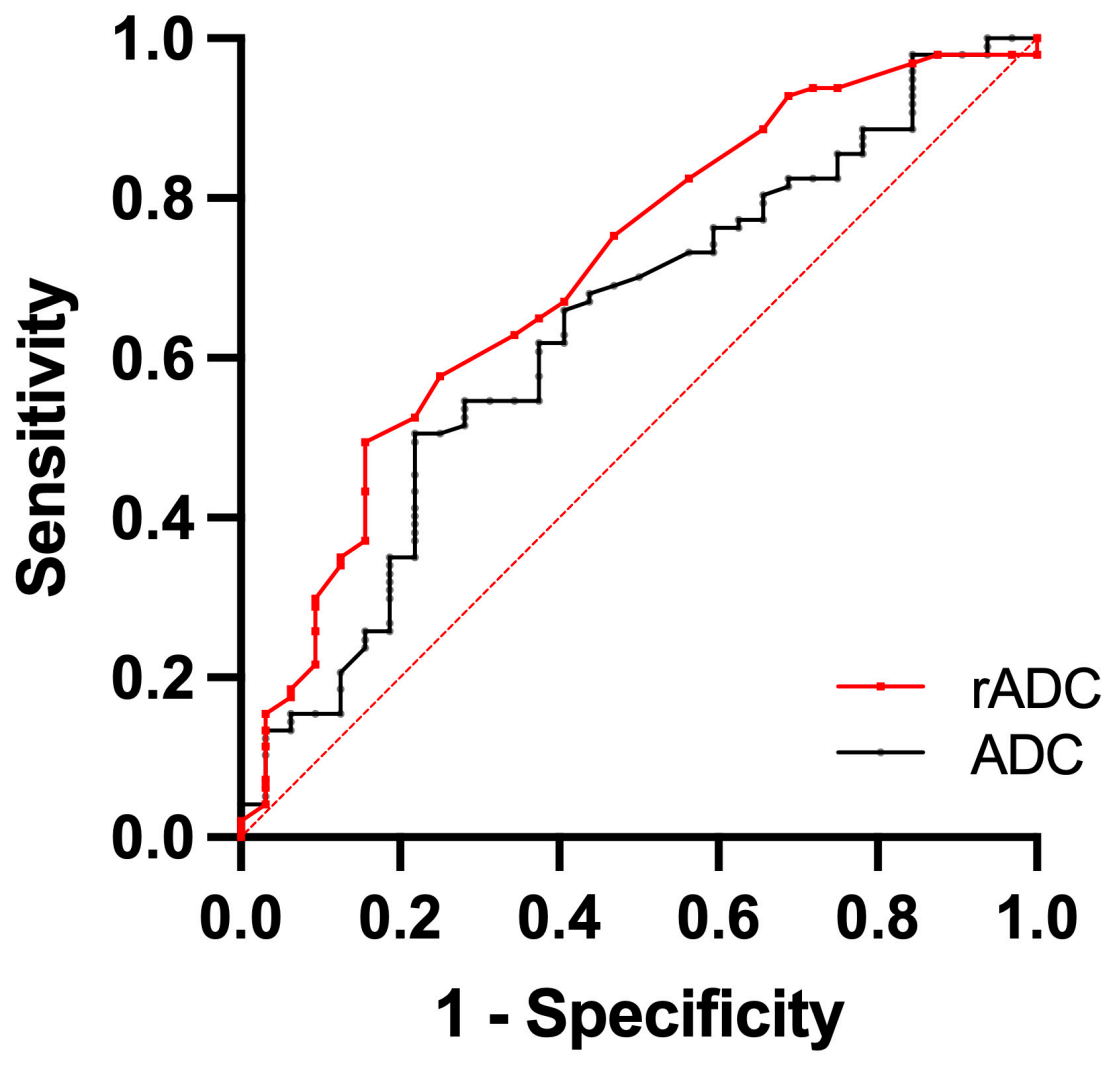
ROC curve of rADC and ADC for predicting END.

Given that both ADC and rADC were significantly associated with END, ROC curve analysis was further performed to evaluate their predictive performance. The rADC demonstrated superior predictive value for END compared with ADC, with AUC values of 0.712 (95% CI: 0.61–0.82, *P* < 0.001) and 0.636 (95% CI: 0.53–0.75, *P* = 0.016), respectively (Figure 3). The optimal cut-off value for rADC was 0.61, yielding a sensitivity of 84% and a specificity of 50%. On multivariable logistic regression analysis incorporating age, sex, maximum infarct diameter, LILI, rADC, and the presence of MCA plaques, both rADC < 0.61 and MCA plaques emerged as independent predictors of END (Table 3).

**Table 3:** Multivariable logistic regression analysis of possible predictors of early neurological deterioration.

Variables	OR (95% CI)	*P-*value
Age	0.99 (0.95–1.03)	0.534
Sex	1.33 (0.53–3.33)	0.542
Maximum infarct diameter	1.13 (0.70–1.81)	0.622
LILI	0.76 (0.49–1.16)	0.202
MCA plaques	2.80 (1.14–6.89)	0.025^*^
rADC < 0.61	4.23 (1.58–11.61)	0.004^*^

OR, odds ratio; CI: confidence interval; LILI, the lowest infarct layer index; MCA, middle cerebral artery; ADC, apparent diffusion coefficient.

^*^Indicates statistical significance.

Among the 129 enrolled patients, 124 (96.1%) underwent conventional MRI within 72 h of symptom onset. A sensitivity analysis was performed in this subgroup to assess the potential influence of MRI timing on ADC measurements. The univariable and multivariable analyses for this subgroup are presented in Supplementary Tables S1, S2, respectively. In the multivariable analysis, rADC < 0.61 and MCA plaques remained independently associated with END, consistent with the findings in the overall cohort.

## Discussion

To our knowledge, this is the first study to comprehensively investigate the relationship between neuroimaging characteristics and END in patients with SSI. Our findings demonstrate that rADC and the presence of MCA plaques are independent predictors of END. Although longitudinal infarct location and maximum infarct diameter were correlated with END on univariate analysis, they did not retain significance as an independent predictor in the multivariate model. In contrast, a higher burden of CSVD was not significantly associated with the occurrence of END in this patient population.

ADC imaging serves as a biomarker of cytotoxic injury, with signal intensity reflecting the degree of lactate production and histological evidence of tissue damage in animal models (24). Oge et al. (25) and Yu et al. (26) both reported that lower ADC values and rADC values were related to the occurrence of END in patients with isolated pontine infarction. In the present study, SSI patients who developed END similarly exhibited significantly lower ADC and rADC values. This concordance may be attributable to the shared pathogenesis between SSI and isolated pontine infarction, as both represent infarctions within perforating artery territories. Lower ADC values likely indicate more severe cytotoxic edema, which may worsen neurological deficits through perilesional compression (27). This is particularly relevant in SSI, given that perforating arteries offer limited collateral compensation, leaving the affected tissue prone to further ischemic damage. Since rADC is referenced to the contralateral mirror region, it is less affected by inter-individual variation in baseline ADC, and thus may offer better reproducibility than ADC (28). This may partly explain its superior predictive performance in our cohort. Therefore, rADC may be a reliable imaging biomarker for early risk stratification in patients with SSI.

MCA plaque was also identified as an independent predictor of END, consistent with previous reports (7, 13). The underlying mechanism may involve additional thrombosis or intraplaque hemorrhage, which could drive the gradual progression from near-occlusion to complete occlusion of the perforating artery, as well as distal embolization from unstable plaques (13, 29, 30). Although both maximum infarct diameter and LILI value were associated with END on univariate analysis, neither retained as independent predictors in the multivariate model. This may be explained from an anatomical perspective, plaques located at the proximal origin of the lenticulostriate arteries tend to cause deeper subcortical infarctions with smaller LILI values while simultaneously producing larger infarcts. Consequently, the association of LILI, and maximum infarct diameter with END may be partly confounded by MCA plaque status, suggesting that MCA plaque represents a more fundamental determinant of neurological deterioration in SSI patients.

The relationship between CSVD burden and END has remained inconclusive. Our study found that CSVD imaging markers were not associated with END in SSI patients. This finding is consistent with Men et al. (31), who demonstrated that culprit plaques of parent arteries, rather than cerebral small vessel damage, could predict END risk in intracranial branch atheromatous disease. However, Jiang et al. (32) identified CSVD burden as an independent predictor of END in SSI patients, which contradicts our results. This discrepancy may be attributable to differences in study populations, as patients in our cohort were relatively younger and presented with a lower CSVD burden. Collectively, these findings further support that intracranial atherosclerosis is the predominant mechanism underlying neurological progression in SSI patients.

Although our study focused on MRI-derived markers, other imaging modalities may also provide prognostic information for END. Previous studies have reported that CT-based early ischemic changes (33, 34) and global cerebral blood flow measured by CT perfusion (35) are associated with END in patients with acute ischemic stroke. Given its wide availability, CT remains the first-line imaging modality in many emergency settings. By contrast, MRI may be more sensitive for detecting small acute infarcts and quantifying early ischemic tissue injury, particularly in SSI, while WB-VWI allows direct assessment of intracranial plaque. Thus, CT- and MRI-derived markers may provide complementary information for END prediction.

Our study has several limitations. First, the retrospective design may have introduced systematic selection bias, potentially affecting the generalizability of our findings. Second, the relatively small sample size and single-center recruitment may have limited statistical powers. Third, this study focused on MRI-derived imaging markers and did not evaluate markers from other imaging modalities. Fourth, the limited field strength and spatial resolution of WB-VWI precluded detection of microatheroma at the proximal LSA origin. Future prospective multicenter studies with larger cohorts are warranted to validate these findings. In addition, integrating multimodal neuroimaging markers into a standardized imaging scoring system may ultimately facilitate early risk stratification in SSI patients.

## Conclusion

Our findings demonstrate that rADC and the presence of MCA plaques are independent predictors of END in SSI patients, whereas CSVD burden was not associated with END. These results help predict clinical progress and guide individualized treatment decisions in patients with SSI.

## Data Availability

The raw data supporting the conclusions of this article will be made available by the authors, without undue reservation.

## References

[1] Duan Z Sun W Liu W Xiao L Huang Z Cao L . Acute diffusion-weighted imaging lesion patterns predict progressive small subcortical infarct in the perforator territory of the middle cerebral artery. Int J Stroke. (2015)10:207–12. doi: 10.1111/ijs.12352 25185717

[2] Yang L Cao W Wu F Ling Y Cheng X Dong Q. Predictors of clinical outcome in patients with acute perforating artery infarction. J Neurol Sci. (2016) 365:108–13. doi: 10.1016/j.jns.2016.03.048 27206885

[3] Takase K Murai H Tasaki R Miyahara S Kaneto S Shibata M . Initial mri findings predict progressive lacunar infarction in the territory of the lenticulostriate artery. Eur Neurol. (2011) 65:355–60. doi: 10.1159/000327980 21625141

[4] Kim SK Song P Hong JM Pak CY Chung CS Lee KH . Prediction of progressive motor deficits in patients with deep subcortical infarction. Cerebrovasc Dis. (2008) 25:297–303. doi: 10.1159/000118373 18303247

[5] Kim JS Yoon Y. Single subcortical infarction associated with parental arterial disease: important yet neglected sub-type of atherothrombotic stroke. Int J Stroke. (2013) 8:197–203. doi: 10.1111/j.1747-4949.2012.00816.x 22568537

[6] Fisher CM. Lacunes: small, deep cerebral infarcts. Neurology. (2011) 77:2104. doi: 10.1212/01.wnl.0000410087.34228.7d 22170942

[7] Yang Y He Y Han W Xu J Cai Z Zhao T . Clinical factors associated with functional outcomes in patients with single subcortical infarction with neurological deterioration. Front Neurol. (2023) 14:1129503. doi: 10.3389/fneur.2023.1129503 37034074 PMC10077891

[8] Song TJ Kim J Song D Yoo J Lee HS Kim YJ . Total cerebral small-vessel disease score is associated with mortality during follow-up after acute ischemic stroke. J Clin Neurol. (2017) 13:187–95. doi: 10.3988/jcn.2017.13.2.187 28406586 PMC5392462

[9] Lau KK Li L Schulz U Simoni M Chan KH Ho SL . Total small vessel disease score and risk of recurrent stroke: validation in 2 large cohorts. Neurology. (2017) 88:2260–7. doi: 10.1212/WNL.0000000000004042 28515266 PMC5567324

[10] Chen Z Li W Sun W Xiao L Dai Q Cao Y . Correlation study between small vessel disease and early neurological deterioration in patients with mild/moderate acute ischemic stroke. Int J Neurosci. (2017) 127:579–85. doi: 10.1080/00207454.2016.1214825 27430627

[11] Rosso C Colliot O Pires C Delmaire C Valabregue R Crozier S . Early adc changes in motor structures predict outcome of acute stroke better than lesion volume. Neuroradiol. (2011) 38:105–12. doi: 10.1016/j.neurad.2010.05.001 20728219

[12] Schaefer PW Ozsunar Y He J Hamberg LM Hunter GJ Sorensen AG . Assessing tissue viability with MR diffusion and perfusion imaging. AJNR Am J Neuroradiol. (2003) 24:436–43. 12637294 PMC7973621

[13] Nam KW Kwon HM Lee YS. Different predictive factors for early neurological deterioration based on the location of single subcortical infarction: early prognosis in single subcortical infarction. Stroke. (2021) 52:3191–8. doi: 10.1161/STROKEAHA.120.032966 34176312

[14] Duan Z Fu C Chen B Xu G Tao L Tang T . Lesion patterns of single small subcortical infarct and its association with early neurological deterioration. Neurol Sci. (2015) 36:1851–7. doi: 10.1007/s10072-015-2267-1 26032577

[15] Nah HW Kang DW Kwon SU Kim JS. Diversity of single small subcortical infarctions according to infarct location and parent artery disease: analysis of indicators for small vessel disease and atherosclerosis. Stroke. (2010) 41:2822–7. doi: 10.1161/STROKEAHA.110.599464 20966406

[16] Zhang C Wang Y Zhao X Wang D Liu L Wang C . Distal single subcortical infarction had a better clinical outcome compared with proximal single subcortical infarction. Stroke. (2014) 45:2613–9. doi: 10.1161/STROKEAHA.114.005634 25052317

[17] Li S Ni J Fan X Yao M Feng F Li D . Study protocol of branch atheromatous disease-related stroke (bad-study): a multicenter prospective cohort study. BMC Neurol. (2022) 22:458. doi: 10.1186/s12883-022-02976-9 36494618 PMC9733351

[18] Li H Dai Y Wu H Luo L Wei L Zhou L . Predictors of early neurologic deterioration in acute pontine infarction. Stroke. (2020) 51:637–40. doi: 10.1161/STROKEAHA.119.027239 31795900

[19] Park TH Lee JK Park MS Park SS Hong KS Ryu WS . Neurologic deterioration in patients with acute ischemic stroke or transient ischemic attack. Neurology. (2020) 95:e2178–91. doi: 10.1212/WNL.0000000000010603 32817184

[20] Staals J Makin SD Doubal FN Dennis MS Wardlaw JM. Stroke subtype, vascular risk factors, and total mri brain small-vessel disease burden. Neurology. (2014) 83:1228–34. doi: 10.1212/WNL.0000000000000837 25165388 PMC4180484

[21] Fazekas F Chawluk JB Alavi A Hurtig HI. Zimmerman RA. MR signal abnormalities at 15 T in Alzheimer's dementia and normal aging. AJR Am J Roentgenol. (1987) 149:351–6. doi: 10.2214/ajr.149.2.351 3496763

[22] Shen M Gao P Zhang Q Jing L Yan H Li H. Middle cerebral artery atherosclerosis and deep subcortical infarction: a 3T magnetic resonance vessel wall imaging study. J Stroke Cerebrovasc Dis. (2018) 27:3387–92. doi: 10.1016/j.jstrokecerebrovasdis.2018.08.013 30145026

[23] Yang T Tang L Jiang S Yan Y Yuan Y Hu Y . A method to distinguish the different etiological mechanisms of single subcortical infarction. Neurol Sci. (2023) 44:1703–8. doi: 10.1007/s10072-023-06623-0 36662315

[24] Neumann-Haefelin T Moseley ME Albers GW. New magnetic resonance imaging methods for cerebrovascular disease: emerging clinical applications. Ann Neurol. (2000) 47:559–70. doi: 10.1002/1531-8249(200005)47:5<559::AID-ANA2>3.3.CO;2-J 10805325

[25] Oge DD Topcuoglu MA Arsava EM. Apparent diffusion coefficient signature of ischemic tissue predicts neurological progression in isolated pontine infarcts. Eur Stroke J. (2022) 7:66–70. doi: 10.1177/23969873211072956 35300260 PMC8921789

[26] Yu W Yang J Liu L Song W Zhang Z Xu M . The value of diffusion weighted imaging in predicting the clinical progression of perforator artery cerebral infarction. Neuroimage Clin. (2022) 35:103117. doi: 10.1016/j.nicl.2022.103117 35872435 PMC9421429

[27] Kim JM Moon J Ahn SW Shin HW Jung KH Park KY. The etiologies of early neurological deterioration after thrombolysis and risk factors of ischemia progression. J Stroke Cerebrovasc Dis. (2016) 25:383–8. doi: 10.1016/j.jstrokecerebrovasdis.2015.10.010 26597264

[28] Koh DM Takahara T Imai Y Collins DJ. Practical aspects of assessing tumors using clinical diffusion-weighted imaging in the body. Magn Reson Med Sci. (2007) 6:211–24. doi: 10.2463/mrms.6.211 18239358

[29] Del Bene A Palumbo V Lamassa M Saia V Piccardi B Inzitari D. Progressive lacunar stroke: review of mechanisms, prognostic features, and putative treatments. Int J Stroke. (2012) 7:321–9. doi: 10.1111/j.1747-4949.2012.00789.x 22463492

[30] Liao S Deng Z Wang Y Jiang T Kang Z Tan S . Different mechanisms of two subtypes of perforating artery infarct in the middle cerebral artery territory: a high-resolution magnetic resonance imaging study. Front Neurol. (2018) 9:657. doi: 10.3389/fneur.2018.00657 30294295 PMC6159754

[31] Men X Hu M Guo Z Li Y Zheng L Wu R . Culprit plaques of large parent arteries, rather than cerebral small vessel disease, contribute to early neurological deterioration in stroke patients with intracranial branch atheromatous disease. Cerebrovasc Dis. (2024) 53:88–97. doi: 10.1159/000530371 36996763

[32] Jiang J Huang X Zhang Y Deng W Shen F Liu J. Total mri burden of cerebral vessel disease correlates with the progression in patients with acute single small subcortical strokes. Brain Behav. (2019) 9:e01173. doi: 10.1002/brb3.1173 30506998 PMC6346414

[33] Dávalos A Toni D Iweins F Lesaffre E Bastianello S Castillo J. Neurological deterioration in acute ischemic stroke: potential predictors and associated factors in the European cooperative acute stroke study (ECASS) I. Stroke. (1999) 30:2631–6. doi: 10.1161/01.STR.30.12.2631 10582989

[34] Grotta JC Welch KM Fagan SC Lu M Frankel MR Brott T . Clinical deterioration following improvement in the NINDS rt-PA Stroke Trial. Stroke. (2001) 32:661–8. doi: 10.1161/01.STR.32.3.661 11239184

[35] Mausbach S Abdallah LA Ben-David E Teitcher M Bornstein NM Eichel RCT. Perfusion imaging as prognostic factor for outcome of lacunar stroke. Neuroradiology. (2024) 66:2223–31. doi: 10.1007/s00234-024-03480-2 39387917 PMC11611928

