# Supplementary material for: Neuroimaging characteristics associated with early neurological deterioration in single subcortical infarction patients
Source: Front Neurol. 2026 Aug 5;17:1849475. doi: 10.3389/fneur.2026.1849475 (PMC13488421; doi:10.3389/fneur.2026.1849475)
Supplement: Supplementary file 1 [file Table_1.docx]

Supplementary Material

Supplementary Table S1. Neuroimaging Characteristics of Patients With and Without END in the Early Conventional MRI Subgroup

| Neuroimaging Characteristics | No-END (92) | END (32) | P |
| --- | --- | --- | --- |
| Infarct volume, cm^3^, median (IQR) | 1.06(0.49–2.46) | 1.39(0.59–2.95) | 0.226 |
| Maximum infarct area on axial, cm^2^, median (IQR) | 0.95(0.52–1.78) | 1.23(0.77–2.31) | 0.073 |
| Maximum infarct diameter on axial, cm, median (IQR) | 1.41(1.08–2.01) | 1.84(1.39–2.20) | 0.011* |
| LILI, median (IQR) | 2(2–3) | 2(1–2) | 0.063 |
| MCA plaques, n (%) | 29(31.5%) | 19(59.4%) | 0.005* |
| ADC of ischemic voxels (mean ± SD) | 485.62 ± 72.66 | 453.22 ±70.02 | 0.030* |
| rADC of ischemic voxels, median (IQR) | 0.61(0.55–0.70) | 0.55(0.51–0.60) | <0.001* |

*SD, standard deviation; IQR, interquartile range; LILI*, *the lowest infarct layer index; ADC, apparent diffusion coefficient; MCA, middle cerebral artery; * Indicates statistical significance.*

Supplementary Table S2. Multivariable Logistic Regression Analysis for END in the Early Conventional MRI Subgroup

|  | OR (95% CI) | P |
| --- | --- | --- |
| Age | 0.99 (0.95–1.03) | 0.524 |
| Sex | 1.41 (0.56–3.58) | 0.466 |
| Maximum infarct diameter | 1.15 (0.72–1.81) | 0.550 |
| LILI | 0.78 (0.50–1.20) | 0.252 |
| MCA plaques | 2.77 (1.10–6.95) | 0.030^*^ |
| rADC<0.61 | 4.04 (1.48–11.05) | 0.006^*^ |

*OR,* odds ratio; *CI:* confidence interval; *LILI,* the lowest infarct layer index; *MCA*, middle cerebral artery; ADC, *apparent diffusion coefficient.* * Indicates statistical significance.
